# Myocardial Fibrosis Quantification Methods by Cardiovascular Magnetic Resonance Imaging in Patients with Fabry Disease

**DOI:** 10.3390/jcm13175047

**Published:** 2024-08-26

**Authors:** Justyna M. Sokolska, Mihály Károlyi, Dana R. Hiestand, Mareike Gastl, Lucas Weber, Mateusz Sokolski, Wojciech Kosmala, Hatem Alkadhi, Christiane Gruner, Robert Manka

**Affiliations:** 1 Institute of Heart Diseases, Faculty of Medicine, Wroclaw Medical University, 50-556 Wroclaw, Poland; justyna.sokolska@umw.edu.pl (J.M.S.); mateusz.sokolski@umw.edu.pl (M.S.);; 2 University Heart Center, Department of Cardiology, University Hospital Zurich, 8091 Zurich, Switzerland; 3 Diagnostic and Interventional Radiology, University Hospital Zurich, University of Zurich, 8091 Zurich, Switzerland; 4 Department of Cardiology, Pulmonology and Vascular Medicine, Heinrich Heine University Düsseldorf, 40225 Düsseldorf, Germany

**Keywords:** Fabry disease, magnetic resonance imaging, cardiomyopathy, cardiac fibrosis

## Abstract

**Background/Objectives**: The presence of late gadolinium enhancement (LGE) on cardiac magnetic resonance (CMR) in patients with Fabry disease (FD) is a predictor of adverse cardiac events. The aim of this study was to establish the most reliable and reproducible technique for quantifying LGE in patients with FD. **Methods**: Twenty FD patients with LGE who underwent CMR on the same scanner and LGE sequence were included. LGE quantifications were done using gray-scale thresholds of 2, 3, 4, 5 and 6 standard deviations (SD) above the mean signal intensity of the remote myocardium, the full width at half maximum method (FWHM), visual assessment with threshold (VAT) and the fully manual method (MM). **Results**: The mean amount of fibrosis varied between quantification techniques from 36 ± 19 at 2SD to 2 ± 2 g using the FWHM (*p* < 0.0001). Intraobserver reliability was excellent for most methods, except for the FWHM which was good (ICC 0.84; all *p* < 0.05). Interobserver reliability was excellent for VAT (ICC 0.94) and good for other techniques (all *p* < 0.05). Intraobserver reproducibility showed the lowest coefficient of variation (CV, 6%) at 5SD and at 2SD and VAT (35% and 38%) for interobserver reproducibility. The FWHM revealed the highest CV (63% and 94%) for both intra- and interobserver reproducibility. **Conclusions**: The available methods for LGE quantification demonstrate good to excellent intra- and interobserver reproducibility in patients with FD. The most reliable and reproducible techniques were VAT and 5SD, whereas the FWHM was the least reliable in the setting of our study. The total amount of LGE varies strongly with the quantification technique used.

## 1. Introduction

Myocardial fibrosis, defined as the presence of late gadolinium enhancement (LGE) in cardiac magnetic resonance (CMR) studies, has been shown to be a valuable marker of unfavorable prognosis in many cardiac diseases, including hypertrophic cardiomyopathy [1,2,3,4,5], aortic stenosis [6,7,8], and dilated cardiomyopathy [9]. Similarly, LGE was found to be a predictor of adverse cardiac events in patients with Fabry disease (FD) [10,11,12], i.e., ventricular arrhythmias, likely induced by a fibrosis substrate. Both qualitative and quantitative assessments of LGE are important for cardiovascular risk stratification; however, diagnostic algorithms addressing this issue have not yet been standardized [13]. 

There are several different quantification techniques available on commercial imaging platforms that are used in daily clinical practice to assess the amount of LGE [14,15]. Nonetheless, there is currently no consensus on which technique is most accurate and should be recommended for specific patient populations [1]. Given the considerable variation of measurements, related to the quantification method used, it is commonly believed that the amount of LGE should be interpreted and/or compared using only the same quantification approach. Importantly, the characteristics and structure of myocardial fibrosis have been shown to vary with the underlying disease [16], which may suggest that there is no single gold standard approach for assessing the amount of LGE on CMR [17,18]. Therefore, LGE quantification approaches should be verified and tested in etiologically homogenous patient populations to ensure that the LGE quantification thresholds are reliably defined and correlated with prognosis.

The aim of this study was to establish the most reliable and reproducible technique for quantifying LGE in patients with FD. 

## 2. Materials and Methods

### 2.1. Study Population

The study population included patients with FD treated in the outpatient clinic at the University Hospital Zurich who underwent CMR between December 2012 and March 2019. In all patients studied, the diagnosis of FD was confirmed by positive genetic testing for an Alpha-galactosidase A gene mutation. 

**Figure 1:**
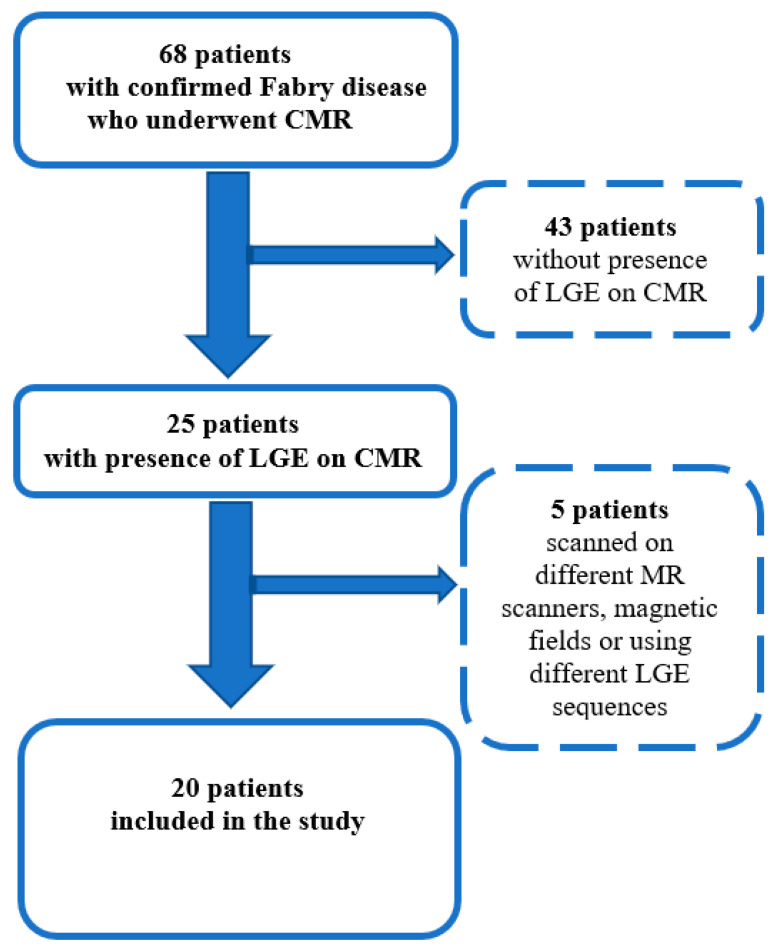
Study flowchart presenting the inclusion and exclusion criteria for the patients with Fabry disease.

To obtain consistent and uniform output data for quantitative LGE assessment, only patients with the presence of LGE who had CMR images with identical LGE sequences and with the same MR scanner were analyzed. Figure 1 shows the study flow chart with the criteria of inclusion and exclusion for patients with FD. 

### 2.2. CMR Imaging Data Acquisition

CMR imaging was performed during breath holding at end-expiration using a 1.5 T MR scanner (Achieva, Philips Healthcare, Best, The Netherlands) with a five-element cardiac phased-array receiver coil. Cine images, including short-axis views covering the entire left ventricle and two-, three- and four-chamber views, were acquired using a balanced steady-state free precession sequence (field of view, 350 × 350 mm^2^; matrix, 200 × 200; repetition/echo time (ms), 3.0/1.5; in-plane resolution, 1.8 × 1.8 mm^2^; number of cardiac phases, 20; slice thickness, 8 mm; and 13–16 contiguous short-axis slices to assess global left ventricular (LV) function). Ten minutes after intravenous administration of 0.2 mmol of gadobutrol (Gadovist; Bayer Schering Pharma, Zurich, Switzerland) per kilogram of body weight, LGE images were acquired in different orientations including short-axis views covering the entire left ventricle and two-, three- and four-chamber views. A two-dimensional LGE technique was used with an inversion recovery gradient-echo MRI sequence (field of view, 350 × 350 mm^2^; matrix, 240 × 240; repetition/echo time (ms), 7.3/4.3; in-plane resolution, 1.5 × 1.5 mm^2^; inversion time individually optimized with a Look–Locker sequence; flip angle, 20 degrees; slice thickness, 8 mm; no gaps; and 13–16 contiguous short-axis slices).

### 2.3. CMR Imaging Data Analysis

**Figure 2:**
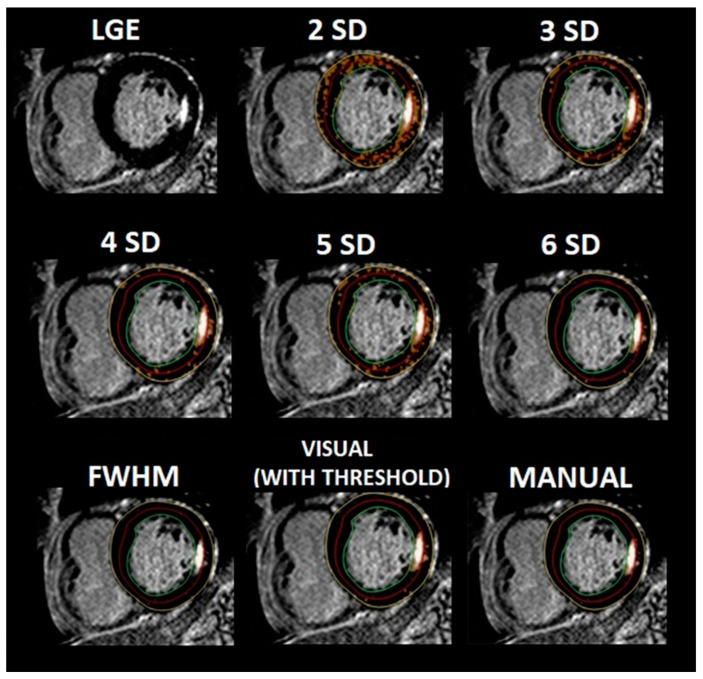
Examples of different methods of late gadolinium enhancement quantification in cardiac magnetic resonance images. Patient with Fabry disease and typical location of myocardial fibrosis in the inferolateral basal segment of the left ventricle. Legend: green line—endocardium; yellow—epicardium; orange—late gadolinium enhancement quantification. Abbreviations: 2SD, 3SD, 4SD, 5SD, 6SD—grayscale thresholds with 2, 3, 4, 5 or 6 (respectively) standard deviations above the mean signal intensity for the remote myocardial tissue; FWHM, full width at half maximum method; LGE, late gadolinium enhancement.

All LGE images were first visually evaluated for the presence of LGE by four observers (two radiologists and two cardiologists), including one with a Level 3 CMR certificate and 18 years of experience in CMR imaging. Only when all four observers agreed on the presence of LGE in the LV myocardium were patients enrolled for further analysis. Endo- and epicardial borders of the LV myocardium were contoured on short-axis LGE images for further LGE quantification. LGE quantifications were performed using grayscale thresholds with 2, 3, 4, 5 and 6 standard deviations (SD) above the mean signal intensity for the remote myocardial tissue, the full width at half maximum (FWHM) method, visual assessment with threshold (VAT) and the fully manual method (MM). Commercially available software (Philips IntelliSpace Portal 10) was used for all CMR analyses. Figure 2 presents contours of the endo- and epicardial borders of the LV myocardium and examples of different techniques of LGE quantification in one patient with FD that was studied. LGE was quantified twice by the same observer at diverse time points (the minimum time period was two weeks) for intraobserver analysis and once by another observer for interobserver analysis. 

### 2.4. Statistical Analysis

The data are presented as means with standard deviations for variables that were normally distributed, as medians with interquartile ranges for skewed variables, and as counts with percentages in cases of categorical variables. The assumption of normality was assessed for all studied parameters using the Kolmogorov–Smirnov test.

The intraclass correlation coefficient (ICC), Bland–Altman analysis and coefficient of variation (CV) were performed to assess intra- and interobserver reliability and reproducibility. An ICC greater than 0.90 was considered to indicate excellent reliability, values between 0.75 and 0.9 indicated good reliability, an ICC between 0.5 and 0.75 indicated moderate reliability, and values less than 0.5 were considered to indicate poor reliability [19].

A value of *p* < 0.05 was considered to indicate statistical significance. Statistical analyses were performed using standard statistical software (Statistica version 13, TIBCO Software Inc., Palo Alto, CA, USA).

## 3. Results

### 3.1. Baseline Characteristics

Sixty-eight patients with FD underwent CMR, 25 of whom had the presence of LGE. Among them, 20 patients were scanned with the same LGE sequence and same MR scanner and were thus included in further analysis (Figure 1). The detailed baseline characteristics of the patients are presented in Table 1.

### 3.2. Amount of LGE

**Figure 3:**
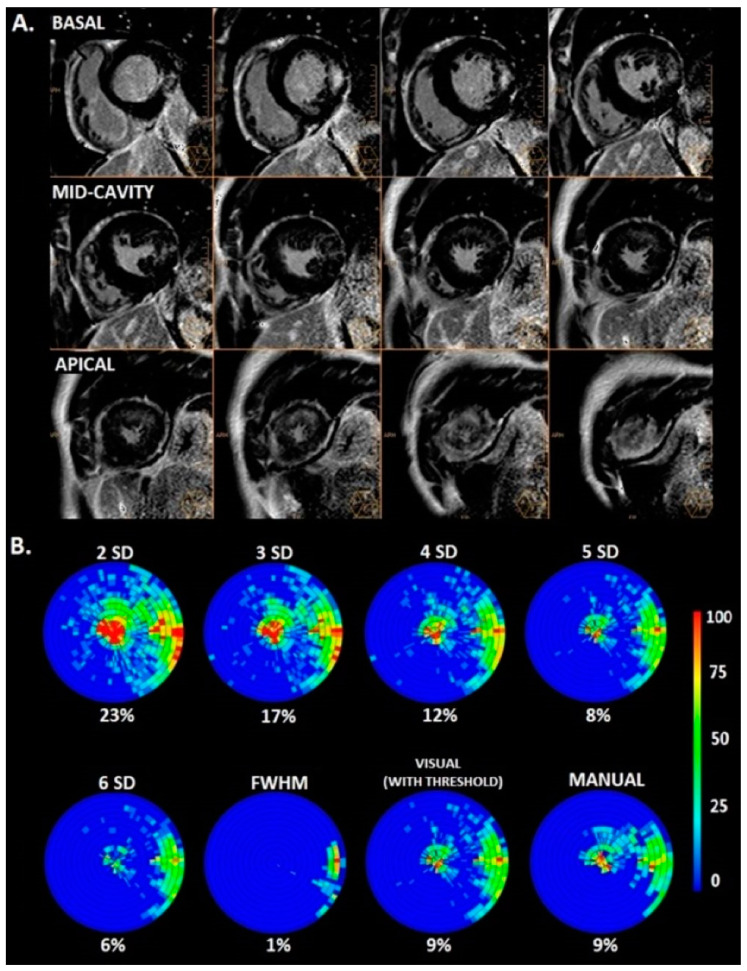
Examples of differences in late gadolinium enhancement quantified by different methods used in cardiac magnetic resonance imaging in patients with Fabry disease. Legend: (**A**) Short-axis cardiac magnetic resonance images with late gadolinium enhancement in a patient with Fabry disease; (**B**) bullseye graphical representation of late gadolinium enhancement quantified by different methods used in the same patient with Fabry disease. The color scale represents the late gadolinium enhanced volume in the left ventricle (percentage). Abbreviations: 2SD, 3SD, 4SD, 5SD, 6SD—grayscale thresholds with 2, 3, 4, 5 or 6 (respectively) standard deviations above the mean signal intensity for the remote myocardial tissue; FWHM, full width at half maximum method.

The mean fibrosis mass (in grams) was 35.5 ± 18.7 at 2SD, 21.0 ± 12.8 at 3SD, 12.7 ± 8.5 at 4SD, 8.0 ± 5.7 at 5SD, 5.3 ± 4.1 at 6SD, 1.9 ± 1.8 using the FWHM, 8.6 ± 7.4 using the VAT and 9.1 ± 6.1 using the MM (*p* < 0.0001). Figure 3 shows an example of the use of different LGE quantification techniques in patients with FD.

### 3.3. Intraobserver and Interobserver Reliability and Reproducibility

The intraobserver reliability of almost all studied LGE quantification methods was excellent, with a range of ICCs from 0.90 for 6SD to 0.95 for VAT, with one exception for the FWHM, which had good intraobserver reliability (ICC = 0.84; all *p* < 0.05).

Interobserver reliability was excellent for VAT (ICC = 0.94) and good for all the other LGE quantification methods (range of ICCs from 0.76 for the MM to 0.87 for 5SD; all *p* < 0.05). The 5SD group had the lowest CV (6%) for intraobserver reproducibility, and the 2SD and VAT groups had the lowest CVs for interobserver reproducibility (35% and 38%, respectively). FWHM had the highest CV for both intra- and interobserver reproducibility (63% and 94%, respectively). 

**Figure 4:**
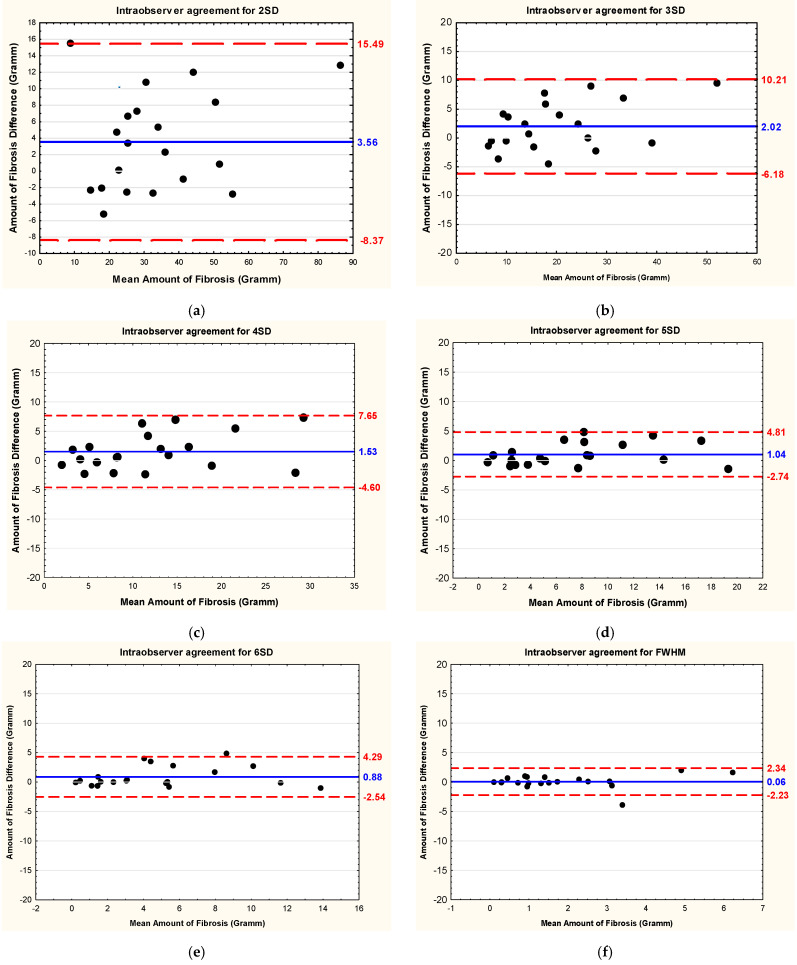

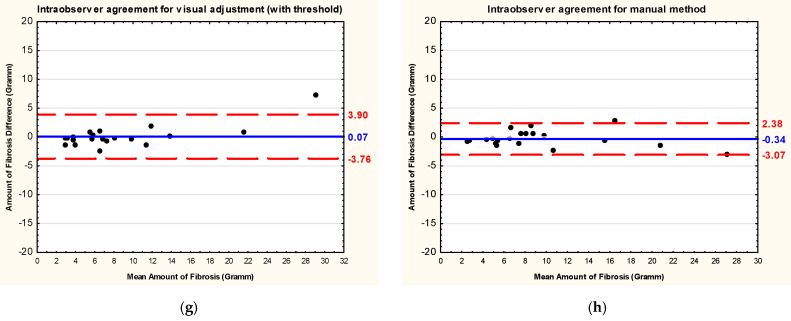
Bland–Altman plots showing intraobserver reproducibility (the mean difference–solid line) of different methods of late gadolinium enhancement quantification used in cardiac magnetic resonance in patients with Fabry disease and corresponding limits of agreement (±1.96 standard deviation—dotted lines) for fibrous masses. (**a**) Method of 2 standard deviations; (**b**) method of 3 standard deviations; (**c**) method of 4 standard deviations; (**d**) method of 5 standard deviations; (**e**) method of 6 standard deviations above the mean signal intensity for the remote myocardial tissue; (**f**) full width at half maximum method; (**g**) method of visual adjustment (with threshold); (**h**) manual method. Abbreviations: 2SD, 3SD, 4SD, 5SD, and 6SD—grayscale thresholds with 2, 3, 4, 5 or 6 (respectively) standard deviations above the mean signal intensity for the remote myocardial tissue; FWHM, full width at half maximum method.

**Figure 5:**
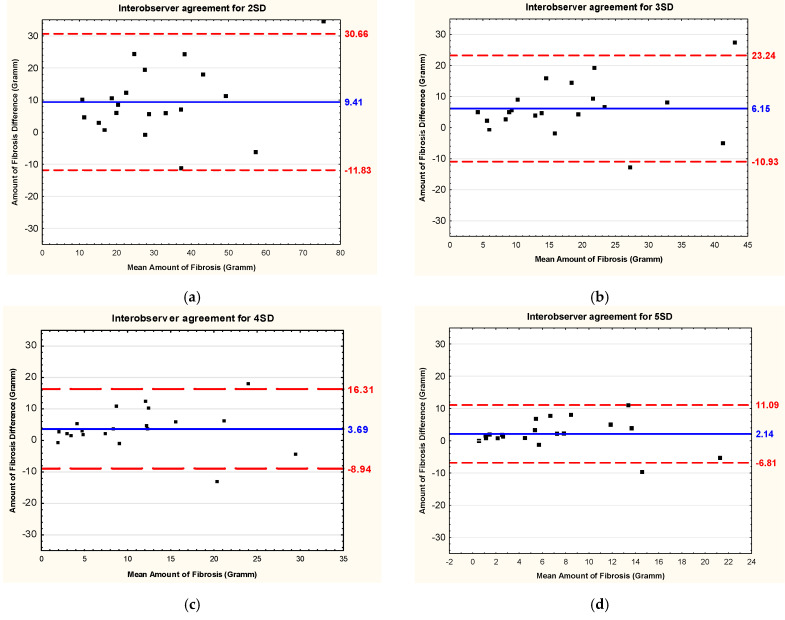

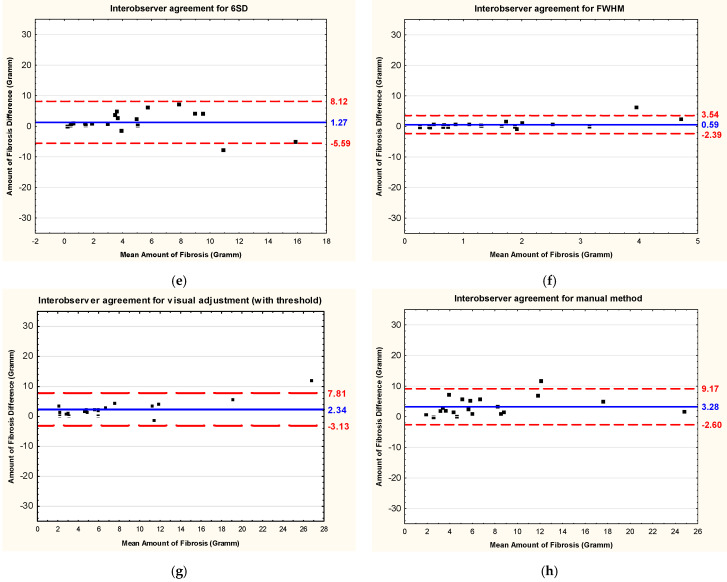
Bland–Altman plots showing interobserver reproducibility (the mean difference–solid line) of different methods of late gadolinium enhancement quantification used in cardiac magnetic resonance in patients with Fabry disease and corresponding limits of agreement (±1.96 standard deviation—dotted lines) for fibrous masses. (**a**) Method of 2 standard deviations; (**b**) method of 3 standard deviations; (**c**) method of 4 standard deviations; (**d**) method of 5 standard deviations; (**e**) method of 6 standard deviations above the mean signal intensity for the remote myocardial tissue; (**f**) full width at half maximum method; (**g**) method of visual adjustment (with threshold); (**h**) manual method. Abbreviations: 2SD, 3SD, 4SD, 5SD, and 6SD—grayscale thresholds with 2, 3, 4, 5 or 6 (respectively) standard deviations above the mean signal intensity for the remote myocardial tissue; FWHM, full width at half maximum method.

The detailed results of the intraobserver and interobserver reliability and reproducibility are shown in Table 2 and Table 3, respectively. Bland–Altman plots showing interobserver and intraobserver reproducibility of the studied methods of LGE quantification, i.e., the mean difference and corresponding 95% limits of agreement for fibrous masses, are presented in Figure 4 and Figure 5, respectively.

**Figure 6:**
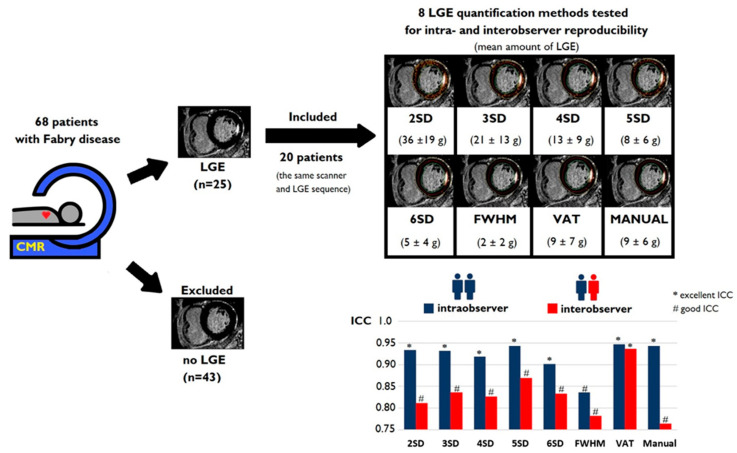
The summary of the studied late gadolinium enhancement quantification methods tested for intra- and interobserver reproducibility in patients with Fabry disease. Abbreviations: 2SD, 3SD, 4SD, 5SD, 6SD—gray-scale thresholds with 2, 3, 4, 5 or 6 (respectively) standard deviations above the mean signal intensity for the remote myocardial tissue; CMR, cardiac magnetic resonance; g, gram; ICC, intraclass correlation coefficient; FWHM, full width at half maximum method; LGE, late gadolinium enhancement; SD, standard deviation; VAT, visual assessment with threshold.

The summary of the obtained study results is presented in Figure 6.

## 4. Discussion

This study demonstrated that the available methods for LGE quantification have good to excellent intra- and interobserver reliability in patients with FD; however, the total amount of LGE varies with the technique used. The best available techniques in the setting of our study (1.5 T MR scanner, a two-dimensional LGE technique) were VAT and 5SD, which had the highest intra- and interobserver ICCs. The FWMH method seems to be inferior to the other techniques because it has the highest CV and the lowest ICC.

In FD, a rare inborn X-linked metabolic disorder, a diminished amount or lack of the enzyme alfa-galactosidase A causes the systemic accumulation of glycosphingolipids. Cardiac involvement may include LV hypertrophy, myocardial inflammation, myocardial fibrosis, heart failure and arrhythmias [20,21]. It has been shown that the maximum benefit of disease-specific therapies is gained by patients who have not yet developed Fabry cardiomyopathy, and with current treatment a limited effect is observed, particularly in patients with the late-onset cardiac variant of FD [21]. Therefore, cardiovascular imaging is crucial for monitoring FD progression and the effects of treatment, with echocardiography and/or CMR repeated every 1–5 years depending on the presence and degree of cardiac abnormalities [20,22]. 

An important part of the CMR examination is myocardial tissue characterization by LGE [23]. In our cohort, LGE was found in 37% of patients with FD, which is consistent with previous data [10]. Histopathological studies have demonstrated that LGE on CMR is associated with focal myocardial collagen scarring [24], the most common of which is located in the inferolateral wall. Myocardial fibrosis in FD usually has a nonischemic pattern, and its patchy and diffuse character makes the precise quantification of LGE challenging [24,25].

The role of LGE quantification in patients with FD has been intensively studied in recent years [10,20]. It has been shown that not only the total mass/volume of LGE, but also the changes in LGE amount over time in patients with FD have prognostic significance, independently determining the arrhythmic, HF and mortality outcomes [10,20]. In our last study on the impact of clinical characteristics and CMR findings on cardiac outcome in patients with FD, we found that both LV hypertrophy and presence of the LGE detected by CMR were associated with adverse cardiac events [12]. In this study, patients were followed up for a median time of 4.9 years. The primary endpoint was composed of cardiac death, new occurrence of atrial fibrillation, heart failure, ventricular tachycardia and bradycardia requiring device implantation. In further quantitative analysis, the amount of LGE was quantified using 6 SD above the mean signal intensity for the normal nulled cardiac muscle and was further analyzed as a percentage of LGE to total LV myocardium. The univariate analysis revealed that the global amount of LGE was associated with an increased risk for the primary endpoint (HR 1.4 per 10% increase in LGE) [12]. This finding supports the need to establish a reliable standard method for LGE quantification in daily clinical practice, as patients with FD usually require serial CMR studies and close follow-up of changes in the total amount of LGE. 

Data assessing the utility of different approaches for LGE quantification in FD are scarce and, to the best of our knowledge, the present study is the first attempt to address this issue in this disease condition. LGE quantification methods have been more extensively studied for other pathologies, such as hypertrophic cardiomyopathy [15,17,26,27]. The common finding is that both in FD and hypertrophic cardiomyopathy the FWHM technique underestimates the total amount of LGE. This observation, together with the fact that the reproducibility of measurements obtained by this technique was inferior to that of other LGE assessment methods, prompted us to postulate that the FWHM may be the least adequate approach for LGE quantification in FD in daily clinical practice. Our findings need to be validated in other FD cohorts; however, existing evidence indicates that there is no single “ideal” technique for LGE quantification and that the available methods cannot be used interchangeably. Therefore, it seems extremely important to report which method was used for myocardial fibrosis quantification in an individual patient with FD and to use the same method for serial LGE assessment.

### Study Limitations

The main limitation of this study is the small sample size. However, given the fact that FD is a very rare condition [21,28] with an estimated prevalence ranging from 1:40,000 to 1:170,000 [29] and that cardiac involvement is present in only half of the patients with FD, we believe that our sample size is sufficient for the purpose of this study.

We do not have a histopathological confirmation of myocardial fibrosis in our patients. However, it has been shown that the presence of LGE is an acceptable noninvasive method for detecting myocardial scarring in both ischemic and nonischemic cardiomyopathies [30,31,32]. 

Another limitation of the presented study is assessment of single CMR scans due to the retrospective nature of this study performed in the setting of standard clinical management in patients with FD. Therefore, the second CMR scans were performed in our cohort usually only after ca. 4 years after the first scan, and during this time some upgrades of scanners, software and even change of MR vendors had occurred, which made a potential comparison follow-up study in the same setting as the first one difficult to perform. In our study, we wanted to establish the most reliable and reproducible technique for quantifying LGE in patients with FD, taking advantage of routine CMR scans performed in this population of patients; therefore, no additional (out of standard of care) CMR scans with contrast were administered for scientific purposes only. However, we believe that our findings are encouraging for the continuation of such studies and should be further validated in a prospective multicenter clinical trial using a bigger cohort of patients with FD with planned follow-up and optimally also recruiting different MR vendors and LGE sequences than those presented in our study.

## 5. Conclusions

All LGE quantification methods studied are characterized by good to excellent intra- and interobserver reliability in patients with FD. The best available techniques in the setting of our study were VAT and 5SD due to their intra- and interobserver reliability being the highest and they might be preferred for LGE quantification in patients with FD. In contrast, compared with other available techniques, the FWHM technique yields the least reproducible results and therefore should be avoided for LGE quantification in FD. The total amount of LGE differed among the LGE quantification methods studied. Hence, in clinical practice, it is crucial to report which technique of LGE quantification was used and the equal approach should be used for follow-up imaging. 

## Figures and Tables

**Table 1:** The baseline characteristics of patients with Fabry disease and the presence of late gadolinium enhancement included in the study.

Patients with Fabry Disease and Presence of LGE Included in the Study n = 20	
Demographics and clinical variables	
Age, years	54 ± 11
Male, n (%)	9 (45)
Angina pectoris symptoms, n (%)	5 (25)
NYHA class I/II, n (%)	14 (70)/6 (30)
ECG-RBBB, n (%)	2 (10)
ECG-LBBB, n (%)	3 (15)
ECG-short PQ, n (%)	2 (10)
Renal involvement, n (%)	15 (75)
Dialysis, n (%)	2 (10)
Kidney transplantation, n (%)	1 (5)
Diabetes, n (%)	0
Coronary heart diseases, n (%)	1 (5)
Arterial hypertension, n (%)	3 (15)
Smoking, n (%)	6 (30)
Laboratory findings	
Troponin level above the normal range, n (%)	16 (80)
NT-proBNP level above the normal range, n (%)	13 (65)
NT-proBNP level, ng/L	563 ± 730 [342, 244–640]
eGFR, mL/min/1.73 m^2^	77 ± 25 [87, 61–92]
Albuminuria, n (%)	15 (75)
Proteinuria, n (%)	10 (50)
Treatment	
Beta-blocker, n (%)	4 (20)
ACEI/ARB, n (%)	10 (50)
Calcium-channel blocker, n (%)	3 (15)
Amiodarone, n (%)	1 (5)
Antiplatelet drug, n (%)	6 (30)
Oral anticoagulation, n (%)	2 (10)
Diuretics, n (%)	0
Aldosterone antagonist, n (%)	0
Statin, n (%)	3 (15)
Antidepressants, n (%)	1 (5)
ERT, n (%)	18 (90)
Duration of ERT, years	5 ± 4
CMR findings	
LVEF, %	69 ± 9
LV maximal wall thickness, mm	13.2 ± 4.7 [12.5, 10.5–14.0]
LV-EDV, mL	137 ± 37
LV-ESV, mL	44 ± 21

Abbreviations: ACEI, angiotensin-converting-enzyme inhibitors; ARB, angiotensin II receptor blocker; CMR, cardiac magnetic resonance; ECG, electrocardiogram; eGFR, estimated glomerular filtration rate; ERT, enzyme replacement therapy; LBBB, left bundle branch block; LGE, late gadolinium enhancement; LV, left ventricle; LV-EDV, left ventricular end-diastolic volume; LV-ESV, left ventricular end-systolic volume; LVEF, left ventricular ejection fraction; n, number of patients; NT-proBNP, N-terminal pro B-type natriuretic peptide; NYHA, New York Heart Association functional classification; PQ, PQ interval; RBBB, right bundle branch block.

**Table 2:** Intraobserver reproducibility of late gadolinium enhancement quantification methods in patients with Fabry disease used in cardiac magnetic resonance.

LGE Quantification Method	Mean Difference	SD	ICC	Lower CI	Upper CI	CV (%)
2 SD	3.56	6.09	0.934 *	0.838	0.974	18.1
3 SD	2.02	4.18	0.932 *	0.833	0.973	20.9
4 SD	1.53	3.12	0.919 *	0.803	0.968	26.0
5 SD	1.04	0.43	0.943 *	0.859	0.978	5.8
6 SD	0.88	0.39	0.901 *	0.763	0.961	8.0
FWHM	0.06	1.17	0.836 *	0.625	0.933	63.0
Visual adjustment (with threshold)	0.07	1.95	0.947 *	0.868	0.979	22.8
Manual	−0.34	1.39	0.943 *	0.859	0.978	15.0

Legend: * indicates a statistically significant difference at *p* < 0.05. Abbreviations: 2SD, 3SD, 4SD, 5SD, 6SD—grayscale thresholds with 2, 3, 4, 5 or 6 (respectively) standard deviations above the mean signal intensity for the remote myocardial tissue; CI, 95% confidence interval; CV, coefficient of variation; ICC, intraclass correlation coefficient; FWHM, full width at half maximum method; LGE, late gadolinium enhancement; SD, standard deviation.

**Table 3:** Interobserver reproducibility of late gadolinium enhancement quantification methods in patients with Fabry disease used in cardiac magnetic resonance.

LGE Quantification Method	Mean Difference	SD	ICC	Lower CI	Upper CI	CV (%)
2 SD	9.41	10.84	0.811 *	0.575	0.923	35.3
3 SD	6.15	8.72	0.836 *	0.625	0.933	48.5
4 SD	3.69	6.44	0.826 *	0.604	0.929	59.1
5 SD	2.14	4.57	0.869 *	0.693	0.947	66.3
6 SD	1.27	3.50	0.833 *	0.619	0.932	74.8
FWHM	0.59	1.50	0.782 *	0.519	0.910	94.2
Visual adjustment (with threshold)	2.34	2.79	0.937 *	0.845	0.975	37.5
Manual	3.28	3.00	0.764 *	0.486	0.902	40.3

Legend: * indicates a statistically significant difference at *p* < 0.05. Abbreviations: 2SD, 3SD, 4SD, 5SD, 6SD—grayscale thresholds with 2, 3, 4, 5 or 6 (respectively) standard deviations above the mean signal intensity for the remote myocardial tissue; CI, 95% confidence interval; CV, coefficient of variation; ICC, intraclass correlation coefficient; FWHM, full width at half maximum method; LGE, late gadolinium enhancement; SD, standard deviation.

## Data Availability

The data underlying this article will be shared upon reasonable request to the corresponding author.
